# Sex Specific Incidence Rates of Type 2 Diabetes and Its Risk Factors over 9 Years of Follow-Up: Tehran Lipid and Glucose Study

**DOI:** 10.1371/journal.pone.0102563

**Published:** 2014-07-16

**Authors:** Arash Derakhshan, Mahsa Sardarinia, Davood Khalili, Amir Abbas Momenan, Fereidoun Azizi, Farzad Hadaegh

**Affiliations:** 1 Prevention of metabolic disorders research center, Research Institute for Endocrine Sciences, Shahid Beheshti University of Medical Sciences, Tehran, Iran; 2 Endocrine Research Center, Research Institute for Endocrine Sciences, Shahid Beheshti University of Medical Sciences, Tehran, Iran; Weill Cornell Medical College - Qatar, Qatar

## Abstract

**Objective:**

To investigate the population-based incidence of type 2 diabetes and its potential risk factors in a sex-split cohort of Iranian population**.**

**Materials and Methods:**

A total of 8400 non-diabetic participants, aged ≥20 years (3620 men and 4780 women) entered the study. Crude and age standardized incidence rates per 1000 person-years were calculated for whole population and each sex separately. Cox proportional hazard models were used to evaluate hazard ratios (HR) and 95% confidence intervals for all potential risk factors in both uni-variable and multivariable models.

**Results:**

During a median follow-up of 9.5 years, 736 new cases of diabetes were identified, including 433 women and 303 men. The annual crude and age-standardized incidence rates (95% CI) of diabetes in the total population were 10.6 (9.92–11.4) and 9.94 (7.39–13.6) per 1000 person-years of follow-up and the corresponding sex specific rates were 10.2 (9.13–11.4) and 9.36 (5.84–14.92) in men and 11.0 (9.99–12.0) and 10.1 (7.24–13.9) in women, respectively. In the multivariable model, the risk for incident diabetes was significantly associated with fasting and 2 hour post challenge plasma glucose as well as family history of diabetes in both men and women. However, among women, only the contribution of wrist circumference to incident diabetes achieved statistical significance [HR: 1.16 (1.03–1.31)] with waist/height ratio being marginally significant [HR: 1.02 (0.99–1.04)]; while among men, only body mass index was a significant predictor [HR: 1.12 (1.02–1.22)]. Additionally, low education level conferred a higher risk for incident diabetes only among men [HR: 1.80 (1.23–2.36); P for interaction with sex = 0.003].

**Conclusion:**

Overall, sex did not significantly modify the impact of risk factors associated with diabetes among Iranian adults; however, among modifiable risk factors, the independent role of lower education and general adiposity in men and central adiposity in women might require different preventive strategies.

## Introduction

Type 2 diabetes is emerging as a modern day epidemic problem that is currently affecting over 370 million adults and this number is expected to reach 500 million by the year 2030 [1]. Above all, aging and increasing rates of urbanization, obesity and physical inactivity are contributing to the rise of diabetes worldwide [2]. Every year over 3.8 million people are dying of diabetes and its complications [3] and several studies have indicated that the risk of cardiovascular diseases (CVD) significantly increases in a glucose intolerant person [4], [5]. In particular, Middle Eastern populations bear the highest burden of diabetes [6]–[9].

Despite horrifying statistics on the population burden of diabetes in the region, data on the dynamics of diabetes among Middle Eastern population continues to be lacking. We have previously reported that sex-adjusted annual incidence rate of diabetes was 1.06% among an Iranian adult population during 6 years of follow-up [9]. Whether the high incidence rates of diabetes alongside the increasing trends of obesity and adoption of sedentary lifestyle [10] has changed or not remains to be elucidated. Moreover, several studies have highlighted the differences between men and women regarding the contribution of risk factors to incident diabetes and the need for sex specific intervention and management strategies [11]–[13]. As a large long-term population–based prospective study, Tehran Lipid and Glucose Study (TLGS) has provided a unique opportunity to assess the incidence of diabetes and its risk factors in the Middle-East region in a sex stratified analysis.

## Methods and Materials

### Study population

TLGS is a dynamic prospective population-based study conducted on a representative sample of Tehranian population with the aim of determining the prevalence of non-communicable disease (NCD) risk factors and developing a healthy lifestyle to improve them. Age distribution of the TLGS population, at baseline, is representative of overall population of Tehran (Iran National Census, 1996). Data collection is ongoing, designed to continue for at least 20 years with about 3-year intervals [14]. Details of the study methods including the recruitment of participants, documentation of medical history and demographic data, clinical examinations, blood sample collections and laboratory and biochemical measurements are explained elsewhere, all of which follow the same method in every phase of TLGS [14].

**Figure 1:**
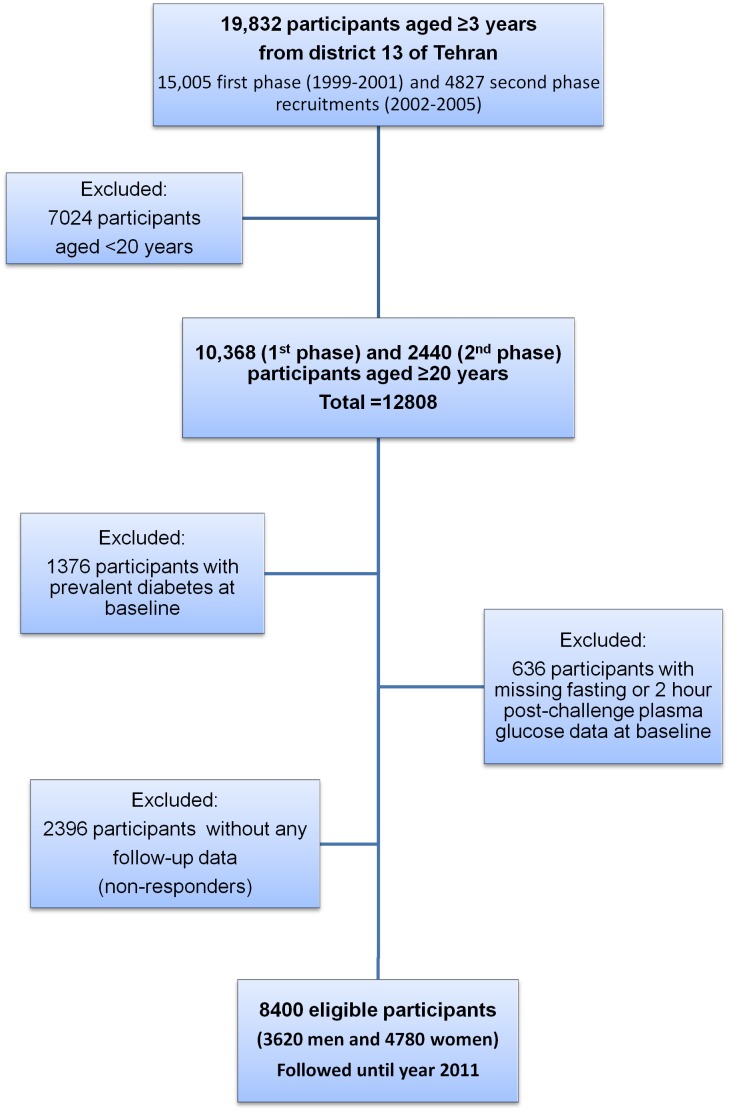
Study population selection flowchart.

To date, it has been conducted in 4 phases on 19,832 participants aged ≥3 years from district 13 of Tehran consisting of 15,005 first phase (1999–2001) and 4827 second phase recruitments (2002–2005). For the current study, 12,808 participants aged ≥20 years at baseline were selected who were recruited from the first and second phase of TLGS. Furthermore, participants with prevalent diabetes (N = 1376) or missing data on fasting and 2 hour post challenge plasma glucose at baseline were excluded (N = 636). Finally, 2396 participants without any follow-up data (non-responders) out of the remaining 10,796 were also excluded which gave us a final number of 8400 participants (3620 men and 4780 women) who were followed until year 2011 (Figure 1). Hence, 77.8% of eligible baseline participants (8400/10796) were entered in the current study.

### Ethics

Informed written consent was obtained from all participants and the Ethical Committee of Research Institute for Endocrine Sciences approved this study.

### Clinical and laboratory measurements

A trained interviewer collected information using a pretested questionnaire. The information obtained included demographic data, drug history, past medical history of CVD, hypertension, and diabetes and smoking status.

Weight was measured, with subjects minimally clothed without shoes, using digital scales (Seca 707: range 0.1–150 kg) and recorded to the nearest 100 g. Height was measured in a standing position without shoes, using a tape meter, while shoulders were in a normal alignment. Waist circumference (WC) was measured at the umbilical level and that of the hip at the maximum level over light clothing, using a tape meter, without any pressure to body surface and measurements were recorded to the nearest 0.1 cm. Wrist circumference was measured to the nearest 0.1 cm using a tape meter. Subjects were asked to hold up the anterior surface of their wrist; the superior border of the tape measure was placed distal to the prominences of radial and ulnar bones then wrist circumference was measured without any pressure over the tape meter. Hip circumference was measured, over light clothing, at the widest girth of the hip. Body mass index (BMI) was calculated as weight in kilograms divided by height in square meters. Waist to height ratio (WHtR) was calculated as WC divided by height (cm).

After a 15-minute rest in the sitting position, two measurements of systolic and diastolic blood pressure (SBP and DBP) were taken, on the right arm, using a standardized mercury sphygmomanometer (calibrated by the Iranian Institute of Standards and Industrial Researches); the mean of the two measurements was considered as the participant’s blood pressure.

A blood sample was taken between 7∶00 and 9∶00 AM from all study participants, after 12 to 14 hours overnight fasting. All the blood analyses were carried out at the TLGS research laboratory on the day of blood collection. Fasting plasma glucose (FPG) and 2-hour post-challenge plasma glucose (2 h-PCPG) were measured using an enzymatic colorimetric method with glucose oxidase; inter- and intra-assay coefficients of variation (CV) at baseline and follow-up phases were both less than 2.3%.

Total cholesterol (TC) was assayed, using the enzymatic colorimetric method with cholesterol esterase and cholesterol oxidase. High density lipoprotein cholesterol (HDL-C) was measured after precipitation of the apolipoprotein B containing lipoproteins with phosphotungistic acid. Triglycerides (TG) were assayed using glycerol phosphate oxidase. Both inter- and intra-assay coefficients of variation were less than 1.9, 3 and 2.1% for TC, HDL-C and TG, respectively, in all baseline and follow-up assays. Analyses were performed using Pars Azmon kits (Pars Azmon Inc., Tehran, Iran) and a Selectra 2 auto-analyzer (Vital Scientific, Spankeren, Netherlands). All samples were analyzed when internal quality control met the acceptable criteria.

Physical activity level was assessed with the Lipid Research Clinic (LRC) questionnaire in the first phase of the TLGS. Due to the inexactness of LRC, it was substituted by the Modifiable Activity Questionnaire (MAQ) from the 2nd phase. This questionnaire measures all three forms of activities including leisure time, job, and household activities in the past year [14].

### Definition of terms

In accordance with the definition provided by the American Diabetes Association, participants were considered to have diabetes if they met at least one of these criteria: FPG ≥7 mmol/L, or 2-h PCPG ≥11.1 mmol/L or taking anti-diabetic medication [15]. In addition, participants with missing data on 2-h PCPG at follow-ups and at the same time having FPG levels<5.05 mmol/L, were considered free of diabetes [16]. A current smoker was defined as a person who smokes cigarettes daily or occasionally. A previous history of CVD reflected any prior diagnosis of CVD by a physician [17]. A positive family history of diabetes was considered as having at least 1 parent or sibling with diabetes mellitus. We defined individuals participating in a vigorous physical activity at least three days per week as physically active. Those participants who entered in the second phase, were considered physically active when achieving a minimum of at least 600 MET (metabolic equivalent task)-minutes per week [18]. Education was classified into 3 groups: Illiterate/primary school, below diploma/diploma and higher than diploma. Participants were also grouped based on being subject to the life-style intervention. Marital status was categorized as single, married and widowed/divorced. Multi-parity was defined as having ≥5 live births.

### Statistical analysis

The participants’ data were split based on sex for all analyses. Baseline characteristics of responders and non-responders (those without any follow-up data) are shown as mean (SD) or frequency (%). Comparisons between responders and non responders were performed using Student’s T-test or χ2 tests as appropriate. Cumulative incidence of diabetes with 95% (CI) was calculated for each sex by dividing the number of new cases of Type 2 diabetes to the total number of subjects in that group. The annualized incidence rate of diabetes was also calculated by dividing the total number of incident cases to the total person-years of follow-up with 95% confidence interval determined by Fisher’s exact test. Age standardized incidence was estimated by direct method using the 2010 Iranian population data from the National Census Bureau. Cox proportional hazards models were used to evaluate associations of potential risk factors with incidence of diabetes in men and women separately. The event date for diabetes cases was described as the middle-time between the date of follow-up visit at which diabetes was detected for the first time, and the most recent follow-up visit preceding the diagnosis; the follow-up time was drawn from the difference between the calculated mid-time date and the date at which the subjects entered the study. For the censored and lost to follow-up subjects, the survival time was the interval between the first and the last observation dates. Follow-up duration and person-years were calculated using the measured survival time.

A uni-variable analysis was performed for each potential risk factor including: Age (years), FPG (mmol/L), 2-h PCPG (mmol/L), BMI (kg/m^2^), WC (cm), WHtR, hip circumference (cm), wrist circumference (cm), SBP (mmHg), DBP (mmHg), TG (mmol/L), HDL-C (mmol/L), TC (mmol/L), family history of diabetes (yes/no), history of CVD (yes/no), smoking status (smoker/non-smoker or past smoker), physically active (yes/no), intervention status (yes/no), education level (higher than diploma (as reference), diploma/cycle, illiterate/primary school), marital status (married (as reference), divorced/widowed, single), multi-parity (yes/no, only for women) and sex (women vs. men). Then, those risk factors with a P value less than 0.2 in uni-variable analysis were selected to enter the multivariable model. In addition, we investigated whether sex modified the relations between potential risk factors and incidence of diabetes. These analyses were performed by producing interaction terms of risk factors with sex in the multivariable model and P values were calculated from likelihood ratio test. Finally, to be comparable with other studies, regardless of any significant interaction between sex and other risk factors, uni-variable and multivariable analyses were also carried out in a pooled data of both sexes. The proportional hazard assumption of the multivariable Cox model was assessed using Schoenfeld’s global test of residuals. All analyses were performed using IBM SPSS for Windows version 19 and STATA version 12 SE (StataCorp LP, TX, USA), with a two-tailed P value<0.05 being considered significant.

## Results

Baseline characteristics of responders and non-responders are illustrated in Table 1. There were significant differences between the TLGS responders (study population) and non-responders in men; responders had higher FPG, BMI, WC, WHtR, TG and TC levels whereas non-responders had higher frequencies of smokers and positive history of CVD. Among women, a few differences were observed which were higher frequencies of smokers and positive history of cardiovascular diseases in non-responders.

**Table 1:** Baseline characteristics of study participants and non-responders (those without follow-up data). Tehran Lipid and Glucose Study 1999–2011.

	Men			Women		
Baseline	Responders	Non- Responders	P value	Responders	Non- Responders	P value
characteristics	N = 3620	N = 1035		N = 4780	N = 1361	
**Age, years**	42.2 (14.6)	41.3 (16.30)	0.14	39.3 (13.1)	39.2 (15.3)	0.85
**FPG, mmol/L**	5.02 (0.52)	4.98 (.52)	0.016	4.91 (.53)	4.92 (.53)	0.67
**2-h PCPG, mmol/L**	5.67 (1.66)	5.64 (1.66)	0.60	6.03 (1.53)	6.09 (1.58)	0.25
**BMI, kg/m^2^**	25.6 (4.07)	25.4 (4.34)	0.07	27.2 (4.89)	27.3 (5.48)	0.59
**WC, cm**	88.6 (11.2)	87.7 (11.7)	0.033	86.3 (12.5)	86.7 (13.3)	0.34
**WHtR**	0.52 (.06)	0.51 (0.07)	0.025	0.55 (.08)	0.55 (.09)	0.17
**Hip, cm**	96.4 (7.11)	96.1 (7.65)	0.30	103.7 (9.49)	103.6 (10.3)	0.80
**Wrist, cm**	17.6 (.95)	17.5 (0.99)	0.14	15.9 (1.04)	15.9 (1.08)	0.44
**SBP, mmHg**	118.7 (16.7)	119.0 (18.4)	0.55	115.4 (17.6)	116.3 (19.5)	0.10
**DBP, mmHg**	76.8 (10.6)	76.9 (11.3)	0.94	76.1 (10.5)	76.3 (11.0)	0.59
**TG, mmol/L**	1.99 (1.35)	1.86 (1.28)	0.006	1.66 (1.01)	1.66 (1.19)	0.96
**HDL-C, mmol/L**	0.98 (0.24)	0.98 (0.24)	0.80	1.16 (.29)	1.16 (.28)	0.52
**TC, mmol/L**	5.17 (1.09)	5.02 (1.08)	<0.001	5.30 (1.21)	5.25 (1.24)	0.18
**FHDM, %**	23.7	24.4	0.63	26.5	24.5	0.12
**CVD Hx., %**	6.3	9.3	0.001	5.5	8.0	0.001
**Smoker, %**	26.4	32.5	<0.001	2.7	5.1	<0.001
**Physically active, %**	26.9	30.1	0.020	28.1	26.2	0.19
**Intervention, %**	42.5	48.7	<0.001	44.4	47.6	0.038
**Education (1), %**	18.6	18.6	0.92	11.1	12.2	0.87
**Education (2), %**	57.3	57.1	-	55.4	53.0	-
**Education (3), %**	24.1	24.3	-	33.5	34.7	-
**Married, %**	80.6	74.4	<0.001	80.8	71.4	<0.001
**Divorced/Widowed, %**	0.6	0.9	-	6.5	8.9	-
**Single, %**	18.7	24.7	-	11.9	18.5	-
**Multi-Parity, %**	-	-	-	16.1	18.8	0.019

BMI, body mass index; WC, waist circumference; WHtR, waist/height ratio; SBP, systolic blood pressure; DBP, diastolic blood pressure; TG, triglycerides; HDL-C, high-density lipoprotein cholesterol ratio; TC, total cholesterol; FHDM, family history of type 2 diabetes; CVD Hx., past history of cardiovascular diseases; Education (1): Higher than diploma, Education (2): Diploma/Cycle, Education (3): Illiterate/Primary. Multi-Parity was defined as having ≥5 live births. Data are means (SD) or proportions. P values for difference between groups were calculated with Student’s T-test or χ2 tests as appropriate.

During a median 9.5 year follow-up (inter-quartile range: 6.13–10.2 years) of 8,400 eligible participants, aged ≥20 (3620 men and 4780 women) of the TLGS, contributing to a total of 68880.54 person-year follow-up, 736 new cases of diabetes were identified, including 433 women and 303 men. The annual crude and age-standardized incidence rate (95% CI) of diabetes in the whole population was 10.6 (9.92–11.4) and 9.94 (7.39–13.6) per 1000 person-years of follow-up and the corresponding sex specific rates were 10.2 (9.13–11.4) and 9.36 (5.84–14.92) in men and 11.0 (9.99–12.0) and 10.1 (7.24–13.9) in women, respectively. The highest rate of incident diabetes was in men aged ≥80 (21.8 per 1000 person-year) and women aged 60–69 (24.0 per 1000 person-year). However, the highest rise in diabetes incidence between age groups was observed in the participants aged 30–39 years compared with those aged 20–29 years, with 2.51 (7.87/3.13) and 2.71 (7.87/2.90) fold increase in men and women respectively (Table 2).

**Table 2:** Diabetes Incidence per 1000 Person-Years by Age and Gender. Tehran Lipid and Glucose study 1999–2011.

Age, year	No. of Participants	Person-Years of Follow-up	Incident Diabetes Cases	Incidence Rate (95%CI) per 1000 Person-Years
**Men**				
** 20–29**	779	6689.2	21	3.13 (1.94–4.79)
** 30–39**	1032	8631.2	68	7.87 (6.11–9.98)
** 40–49**	716	5928.0	76	12.8 (10.1–16.0)
** 50–59**	504	3968.9	64	16.1 (12.4–20.5)
** 60–69**	438	3345.4	56	16.7 (12.6–21.7)
** 70–79**	136	877.3	16	18.2 (10.4–29.6)
** 80≤**	15	91.6	2	21.8 (2.64–78.8)
** Total**	3620	29531.9	303	10.2 (9.13–11.4)†
				9.36 (5.84–14.92)^‡^
**Women**				
** 20–29**	1311	11013.8	32	2.90 (1.98–4.10)
** 30–39**	1363	11684.5	92	7.87 (6.34–9.65)
** 40–49**	985	8111.3	117	14.4 (11.9–17.2)
** 50–59**	668	5232.9	117	22.3 (18.4–26.8)
** 60–69**	376	2748.6	66	24.0 (18.5–30.5)
** 70–79**	74	532.6	9	16.9 (7.72–32.0)
** 80≤**	3	24.6	0	-
** Total**	4780	39348.5	433	11.0 (9.99–12.0)†
				10.1 (7.24–13.9)^‡^
**Total**				
** 20–29**	2090	17703.1	53	2.99 (2.24–3.91)
** 30–39**	2395	20315.8	160	7.87 (6.70–9.19)
** 40–49**	1701	14039.3	193	13.75 (11.8–15.8)
** 50–59**	1172	9201.9	181	19.6 (16.9–22.7)
** 60–69**	814	6094.1	122	20.0 (16.6–23.9)
** 70–79**	210	1409.9	25	17.3 (11.4–26.1)
** 80≤**	18	116.2	2	17.2 (2.08–62.1)
** Total**	8400	68880.5	736	10.6 (9.92–11.4)†
				9.94 (7.39–13.6)^‡^

† Crude incidence rate. **^‡^**Age standardized incidence rate based on the 2010 Iranian population data from the National Census Bureau. 95% Confidence intervals (CI) were calculated using Fisher’s exact test.

Table 3 presents the uni-variable contribution of each candidate predictor to the risk of developing incident diabetes, separately for men and women. All potential clinical and laboratory risk factors were significantly related to incident diabetes in both sexes and in total. However, past history of CVD [HR (95% CI): 2.29 (0.73–7.19)] and HDL-C level [HR (95% CI): 0.62 (0.38–1.01)] had no association with incidence of diabetes among men. In addition, in the pooled uni-variable analysis, sex was not a risk factor for incident diabetes [(HR (95% CI) for women vs. men: 1.06 (0.92–1.23), p value = 0.39].

**Table 3:** Hazard Ratios (HR) and 95% Confidence Intervals (CI) from the Uni-variable Analysis of Potential Risk Factors in Relation to Diabetes Incidence by Gender.

Risk Factors	Men	P value	Women	P value	Total	P value
**Age, years**	1.03 (1.02–1.04)	<0.001	1.04 (1.03–1.05)	<0.001	1.03 (1.03–1.04)	<0.001
**FPG, mmol/L**	6.78 (5.63–8.17)	<0.001	7.32 (6.29–8.52)	<0.001	7.01 (6.23–7.88)	<0.001
**2-h PCPG, mmol/L**	1.76 (1.66–1.87)	<0.001	1.90 (1.80–2.01)	<0.001	1.84 (1.76–1.91)	<0.001
**BMI, kg/m^2^**	1.12 (1.10–1.15)	<0.001	1.13 (1.11–1.15)	<0.001	1.13 (1.11–1.14)	<0.001
**WC, cm**	1.05 (1.04–1.06)	<0.001	1.06 (1.05–1.07)	<0.001	1.06 (1.05–1.06)	<0.001
**WHtR**	1.09 (1.08–1.11)	<0.001	1.10 (1.08–1.11)	<0.001	1.09 (1.08–1.10)	<0.001
**Hip, cm**	1.05 (1.04–1.07)	<0.001	1.04 (1.03–1.05)	<0.001	1.04 (1.03–1.05)	<0.001
**Wrist, cm**	1.62 (1.44–1.82)	<0.001	1.69 (1.56–1.83)	<0.001	1.36 (1.28–1.44)	<0.001
**SBP, mmHg**	1.02 (1.01–1.02)	<0.001	1.02 (1.02–1.03)	<0.001	1.02 (1.02–1.03)	<0.001
**DBP, mmHg**	1.04 (1.03–1.05)	<0.001	1.04 (1.03–1.05)	<0.001	1.04 (1.03–1.05)	<0.001
**TG, mmol/L**	1.24 (1.19–1.29)	<0.001	1.42 (1.35–1.50)	<0.001	1.28 (1.24–1.32)	<0.001
**HDL-C, mmol/L**	0.62 (0.38–1.01)	0.059	0.40 (0.28–0.57)	<0.001	0.52 (0.39–0.68)	<0.001
**TC, mmol/L**	1.38 (1.26–1.52)	<0.001	1.37 (1.28–1.46)	<0.001	1.37 (1.30–1.45)	<0.001
**FHDM**	1.79 (1.41–2.26)	<0.001	2.01 (1.66–2.44)	<0.001	1.92 (1.66–2.23)	<0.001
**CVD Hx.**	2.29 (0.73–7.19)	0.153	2.16 (1.60–2.93)	<0.001	2.18 (1.64–2.91)	<0.001
**Smoking**	0.97 (0.75–1.26)	0.840	0.96 (0.53–1.76)	0.91	0.94 (0.75–1.18)	0.61
**Physically active**	0.97 (0.74–1.27)	0.849	0.97 (0.78–1.20)	0.78	0.97 (0.82–1.15)	0.76
**Intervention**	1.02 (0.81–1.29)	0.829	1.03 (0.85–1.25)	0.71	1.03 (0.89–1.19)	0.66
**Education (1)**	Reference	-	Reference	-	Reference	-
**Education (2)**	2.50 (1.71–3.64)	<0.001	2.54 (1.75–3.67)	<0.001	2.62 (2.02–3.40)	<0.001
**Education (3)**	1.45 (1.01–2.08)	0.04	0.90 (0.61–1.31)	0.58	1.14 (0.87–1.48)	0.32
**Married**	Reference	-	Reference	-	Reference	-
**Divorced/Widowed**	1.50 (0.48–4.68)	0.48	1.46 (1.07–2.00)	0.015	1.45 (1.08–1.95)	0.012
**Single**	0.24 (0.15–0.40)	<0.001	0.22 (0.13–0.39)	<0.001	0.23 (0.16–0.34)	<0.001
**Multi-Parity**	-	-	2.41 (1.96–2.96)	<0.001	-	-
**Sex, Women**	-	-	-	-	1.06 (0.92–1.23)	0.39

Tehran Lipid and Glucose study 1999–2011.

BMI, body mass index; WC, waist circumference; WHtR, waist/height ratio; SBP, systolic blood pressure; DBP, diastolic blood pressure; TG, triglycerides; HDL-C, high-density lipoprotein cholesterol ratio; TC, total cholesterol; FHDM, family history of type 2 diabetes; CVD Hx., past history of cardiovascular diseases; Education (1): Higher than diploma, Education (2): Diploma/Cycle, Education (3): Illiterate/Primary. Multi-Parity was defined as having ≥5 live births. Cox proportional hazards models were used to calculate HRs and 95% CIs.

Contributions of all important risk factors to incident diabetes in the multivariable model are illustrated in Table 4. In the multivariate analysis, we observed that the risk of incident diabetes was significantly related with FPG and 2 h-PCPG levels among men and women and family history of diabetes was also independently associated with diabetes incident in both sexes.

**Table 4:** Hazard Ratios and 95% Confidence Intervals (CI) from the Multi-variable Analysis of Potential Risk Factors in Relation to Diabetes Incidence by Gender.

Risk Factors	Men	P value	Women^†^	P value	Total	P value
**Age, years**	1.01 (0.99–1.02)	0.16	0.99 (0.98–1.00)	0.54	1.00 (0.99–1.01)	0.92
**FPG, mmol/L**	3.30 (2.65–4.10)	<0.001	3.54 (2.94–4.26)	<0.001	3.39 (2.93–3.91)	<0.001
**2-h PCPG, mmol/L**	1.43 (1.34–1.54)	<0.001	1.43 (1.34–1.53)	<0.001	1.42 (1.35–1.49)	<0.001
**BMI, kg/m^2^**	1.12 (1.02–1.22)	0.01	1.03 (0.97–1.09)	0.21	1.04 (0.99–1.10)	0.07
**WHtR**	0.97 (0.93–1.02)	0.35	1.02 (0.99–1.05)	0.05	1.02 (0.99–1.04)	0.06
**Hip, cm**	0.97 (0.94–1.00)	0.13	0.98 (0.96–1.00)	0.13	0.98 (0.97–1.00)	0.11
**Wrist, cm**	1.07 (0.91–1.26)	0.36	1.16 (1.03–1.31)	0.01	1.07 (1.00–1.16)	0.04
**SBP, mmHg**	1.00 (0.99–1.00)	0.93	1.00 (0.99–1.01)	0.13	1.00 (0.99–1.00)	0.53
**DBP, mmHg**	1.01 (0.99–1.02)	0.11	1.00 (0.99–1.01)	0.68	1.00 (0.99–1.01)	0.23
**TG, mmol/L**	1.06 (0.99–1.13)	0.06	1.02 (0.93–1.12)	0.60	1.05 (0.99–1.10)	0.06
**HDL-C, mmol/L**	1.00 (0.57–1.74)	0.99	0.81 (0.53–1.23)	0.33	0.90 (0.65–1.25)	0.55
**TC, mmol/L**	0.99 (0.88–1.11)	0.89	0.94 (0.85–1.04)	0.23	0.97 (0.90–1.05)	0.51
**FHDM**	1.78 (1.39–2.29)	<0.001	1.54 (1.26–1.89)	<0.001	1.64 (1.40–1.92)	<0.001
**CVD Hx.**	1.86 (0.58–5.97)	0.29	1.09 (0.78–1.52)	0.60	1.27 (0.92–1.76)	0.14
**Education (1)**	Reference	-	Reference	0.10	Reference	-
**Education (2)**	1.46 (0.96–2.23)	0.07	0.92 (0.60–1.41)	0.72	1.28 (0.95–1.72)	0.09
**Education (3)**	1.80 (1.23–2.63)	0.002	0.73 (0.48–1.09)	0.12	1.25 (0.95–1.65)	0.10
**Married**	Reference	0.67	Reference	0.62	Reference	-
**Divorced/Widowed**	1.03 (0.30–3.47)	0.95	1.01 (0.72–1.41)	0.94	1.66 (0.78–3.52)	0.18
**Single**	0.78 (0.45–1.34)	0.37	0.73 (0.39–1.37)	0.33	0.75 (0.50–1.12)	0.16
**Multi-Parity**	-	-	1.00 (0.77–1.29)	0.97	-	-

Tehran Lipid and Glucose study 1999–2011.

BMI, body mass index; WC, waist circumference; WHtR, waist/height ratio; SBP, systolic blood pressure; DBP, diastolic blood pressure; TG, triglycerides; HDL-C, high-density lipoprotein cholesterol ratio; TC, total cholesterol; FHDM, family history of type 2 diabetes; CVD Hx., past history of cardiovascular diseases; Education (1): Higher than diploma, Education (2): Diploma/Cycle, Education (3): Illiterate/Primary. Multi-Parity was defined as having ≥5 live births. ^†^HRs are from the multi-variable model containing marital status; HR for Multi-Parity was calculated in a separate model without marital status because of their co-linearity, this did not affect HRs of other variables (Data not shown). Cox proportional hazards models were used to calculate HRs and 95% CIs.

It was only among women that the contribution of wrist circumference to incident diabetes achieved statistical significance, [HR (95% CI): 1.16 (1.03–1.31)], while its predictability faded in multivariate model among men [HR (95% CI): 1.07 (0.91–1.26)]. On the other hand, BMI was independently associated with the incidence of diabetes only among men [HR (95% CI): 1.12 (1.02–1.22) vs. women [HR (95% CI): 1.03 (0.97–1.09)]. Among all covariates, education level of illiterate/primary school had a significant interaction with sex in the multivariate model (P value = 0.003). Accordingly, among men the lowest education level (illiterate/primary school) was associated with a higher risk of diabetes incidence, [HR (95% CI): 1.80 (1.23–2.36)], while this association was not significant among women, [HR (95% CI): 0.73 (0.48–1.09)].

Finally, in the pooled multivariable analysis (which did not include sex because of a P value>0.2 in the uni-variable model); FPG, 2 h-PCPG, wrist circumference and family history of diabetes were significant risk factors for incident diabetes. Furthermore, WHtR and BMI had a marginally significant association with diabetes incidence [HR (95% CI): 1.02 (0.99–1.04) and 1.04 (0.99–1.10), respectively].

## Discussion

To the best of our knowledge, the current study is the first prospective population-based study to examine the sex-specific incidence of type 2 diabetes in a Middle Eastern population characterized by a relatively high incidence of obesity and metabolic syndrome [10], [19]. In this community-based prospective cohort study of adult Iranian men and women, during 9 years of follow-up the age-standardized incidence rates (95% CI) of diabetes were 9.36 (5.84–14.92) in men and 10.1 (7.24–13.9) in women. Moreover, the role of different modifiable risk factors in progression to diabetes was investigated. Our study demonstrated strong associations of fasting and post-challenge glucose levels and family history of diabetes with incidence of the disease in both sexes. However, among anthropometric measures, only BMI in men and wrist circumference in women were related to incident diabetes. Another key finding was the association of low education (as a socioeconomic factor) to the incidence of diabetes, solely among men.

Data on the incidence rate of diabetes for Middle-East populations is really scarce, especially from a long-term cohort with repeated clinical and laboratory evaluations. TLGS have previously reported an age and sex standardized incidence rate of 10.6 per 1000 person-years among 3307 participants, aged ≥20 years after a median follow-up of 6 years [9]. The age-standardized incidence rate in the pooled population of this study is lower than the previous study (9.94 (7.39–13.6) versus 10.6 (9.2–12.1) per 1000 person-years). However, these two studies are not exactly comparable because of the different population size and inclusion of 2^nd^ phase recruits, longer follow-up duration, and sex specified analyses of this study. It is important to realize that in the current study, by applying survival analysis, data of healthy censored participants during each stage of the follow-up were also accounted for which results in a more precise estimation of diabetes incidence.

Crude and age-standardized incidence rates of diabetes widely differ in different populations. This fact might be due to variation in genetic predisposition, characteristics, methodological assessment procedures and also different extent of exposure to diabetes risk factors in various populations. The high incidence of type 2 diabetes in this population is higher than several rates reported around the globe. Considering studies which reported whole population data without splitting by sex, the pooled incidence rate of diabetes was reported to be 9.6 per 1000 person-years in Japan [20], 7.6 per 1000 person-years in the Bruneck study of Italy [21], 10.8 per 1000 person-years in the Asturias Study of Spain [22] and 5.15 per 1000 population in the United Kingdom [23]; however, the reported incidence rate of diabetes in an urban normal glucose tolerant population of India was 20.2 per 1000 person years which is extremely higher than our population [24]. Indeed, as previously reported in TLGS, during 1999–2008, the prevalence of obesity increased 33 and 23% in men and women respectively, and abdominal obesity during this period showed an increase of 71% in men and 9% in women [10]. Thus, the reported incidence rate of type 2 diabetes might be attributable to the alarming rise of obesity and metabolic syndrome in our population [19] and the impact of urbanization resulting in a sedentary lifestyle and unhealthy diet [25]. Furthermore the high incidence of diabetes in our population might also be a result of ethnicity and air pollution [26], [27]. Most important of all, the highest rise in diabetes incidence in comparison with the previous age group occurred in participants aged 30–39 years. This alarming finding signifies that prevention strategies and interventions should target the population at a younger age (20–29 years old).

There are few studies that consider the incidence of type 2 diabetes in each sex separately. In a study by Hippisley-Cox et al in England and Wales, the age standardized rates (95% CI) were 5.31 (5.26 to 5.36) per 1000 person years for men and 4.13 (4.08–4.17) per 1000 person years for women for the white ethnicity group; the equivalent rates for Bangladeshi men was 19.34 (14.28–24.4) and for Bangladeshi women 18.20 (12.93–23.47) per 1000 person years; For Pakistanis, the age standardized rates were 13.22 (11.24–15.21) for men 11.19 (9.16–13.21) for women [28]. Additionally, Meisinger et al. in the MONICA study reported the age-standardized incidence rates of 5.8 per 1000 person-years for men and 4.0 per 1000 person- years for women [11]. All of the mentioned results show the diversity of diabetes incidence among different ethnicities, with remarkably higher rates among Middle-Eastern and south Asian populations.

Consistent with results of many other studies [29]–[32], we demonstrated that FPG, 2-h PCPG and family history of diabetes is highly predictive of the disease in both sexes; however, age was not associated with progression to diabetes which might be due to the mediation effects caused by age-dependency of anthropometric measures, SBP, TG and HDL-C [33]–[37].

Different anthropometric measures were examined in relation to incident diabetes. Surprisingly, only BMI in men and wrist circumference as well as WHtR (marginally significant) in women were related to the development of the disease. Some studies have suggested that central adiposity has stronger association with diabetes incidence than general obesity [38]–[40]. There is discrepancy about which measure would better predict risk of type 2 diabetes. While two previous meta-analyses found no clear differences between BMI and WC as predictors of diabetes [38], [41] a recent meta-analysis claimed that WHtR would show a superior predictive ability than BMI or WC [42].

Results of this study suggested that there were sex differences regarding the relation of anthropometric measures with incident diabetes. Similar to our results, Meisinger et al. in the MONICA/KORA Augsburg Cohort Study [43], Li et al. in the United States Third National Health and Nutrition Examination Survey [44] and Schulze et al. in the EPIC-Potsdam study [45] have all highlighted that central adiposity measurements perform better than BMI for diabetes prediction in women whereas there is not much difference between central adiposity indices and BMI in relation with incident diabetes in men. In addition, Langenberg et al. in The EPICInterAct Case-Cohort Study have suggested that that WC while adjusted with general adiposity might be a better determinant of abdominal fat and diabetes risk in women, because women’s WC is mostly determined by subcutaneous fat compared to men [46]. It must be noted that in the present study, the variations of hip circumference has also been accounted for and because a larger hip circumference is protective against diabetes development especially in women [47], [48]; therefore, the role of central adiposity in association with diabetes has become more prominent in women. Moreover, in the meta-analysis by Kodama et al. it was suggested that in the populations with a rather high BMI no difference may be observed between adiposity indices in relation with incident diabetes [42], the issue that might be true for our women population with a mean baseline BMI of about 27.2 kg/m^2^. Finally, to the best of our knowledge this is the first study that examined the impact of four different anthropometric measures together in a sex split analysis in association with diabetes incidence; meaning that the effects of general, central and gluteal adiposity as well as bone size and body frame (measured by wrist circumference) altogether have been taken into account, while no co-linearity was observed between these measures.

Similar to a recent TLGS study [49], wrist circumference was related to incident diabetes only in women. The reason for the differences between sexes could be explained by the effects of sex steroid hormones and their interaction with bone metabolism and glucose homeostasis [49].

Another interesting finding was the association of low education level with incident diabetes in men. The impact of lower education on incident diabetes as suggested by Williams et al. in the Australian Diabetes Obesity and Lifestyle Study might be partly mediated by smoking and physical activity [50]. On the contrary, in two sex specific studies in the United States and Sweden, low education was associated with diabetes in women [51], [52].

It has been highlighted that among high risk adults moderate physical activity reduces risk of progression to diabetes, with a greater risk reduction if followed by weight loss. However, a moderate physical activity (150 minutes per week) did not prevent all diabetes especially among those with high baseline risk of the disease [53]. In the current study we did not find a significant association between diabetes incidence and baseline physical activity even in uni-variable analysis. This might be due to the high prevalence of physical inactivity in the TLGS and national Iranian population [54], which may cause a loss of association between physical activity and incident diabetes because of lack of variation. Secondly, our questionnaires used to evaluate physical activity might not provide complete data accurately considering that physical activity is an important but difficult to measure variable with adequate precision [55]. Third, because of a high prevalence of cardiometabolic risk factor among Iranian population (including general and central adiposity as well as dyslipidemia) [56]; as suggested by Gill and Cooper [53], the intensity of physical activity needed to reduce diabetes risk might be higher than that of suggested by the guidelines [15].

The strengths of the present prospective study could be the reasonable size of population, length of follow-up and using the direct measurements of the anthropometric indices rather than self-reported data. Furthermore, several potential and known risk factor of diabetes were examined and the sex specific method of this study adds to the understanding of the different contribution of risk factors to the development of diabetes in men and women.

Some limitations of the current study are needed to be addressed. Firstly, men who responded to the follow-up examinations of TLGS had a relatively high FPG (5.02 vs. 4.98 mmol/l) and higher WC (88.6 vs. 87.7 cm) as well as higher TG (1.99 vs. 1.86 mmol/l) compared with non-responders; however, the responders reported a lower frequency of CVD history (6.3% vs. 9.3%) and lower rate of smokers (26.4% vs. 32.5%); therefore, results of this study might be an overestimation of the true diabetes incidence rate in our men population. Secondly, this study has been conducted on a sample of Iranian population and further studies should be conducted to determine whether our findings are applicable to other populations of Middle East.

In summary, type 2 diabetes currently represents a huge global public health problem. We estimated the age-standardized incidence rates of diabetes to be 9.36 (5.84–14.92) in men and 10.1 (7.24–13.9) in women. We also observed that diabetes incidence was strongly associated with FPG, 2 h-PCPG and family history of diabetes in both genders. However, only BMI and lower education among men and WHtR and wrist circumference among women were related to incident diabetes. Overall, sex did not significantly modify the impact of risk factors associated with diabetes among Iranian adults; however, among modifiable risk factors, the independent role of lower education and general adiposity in men and central adiposity in women might require different preventive strategies.
